# Expanding the Cyanobacterial Nitrogen Regulatory Network: The GntR-Like Regulator PlmA Interacts with the PII-PipX Complex

**DOI:** 10.3389/fmicb.2016.01677

**Published:** 2016-10-28

**Authors:** Jose I. Labella, Anna Obrebska, Javier Espinosa, Paloma Salinas, Alicia Forcada-Nadal, Lorena Tremiño, Vicente Rubio, Asunción Contreras

**Affiliations:** ^1^Departamento de Fisiología, Genética y Microbiología, Universidad de AlicanteAlicante, Spain; ^2^Instituto de Biomedicina de Valencia of the CSICValencia, Spain; ^3^Group 739, CIBER de Enfermedades Raras (CIBERER-ISCIII)Valencia, Spain

**Keywords:** PlmA, PII, PipX, cyanobacteria, nitrogen regulation, signaling, GntR, three hybrid interactions

## Abstract

Cyanobacteria, phototrophic organisms that perform oxygenic photosynthesis, perceive nitrogen status by sensing 2-oxoglutarate levels. PII, a widespread signaling protein, senses and transduces nitrogen and energy status to target proteins, regulating metabolism and gene expression. In cyanobacteria, under conditions of low 2-oxoglutarate, PII forms complexes with the enzyme N-acetyl glutamate kinase, increasing arginine biosynthesis, and with PII-interacting protein X (PipX), making PipX unavailable for binding and co-activation of the nitrogen regulator NtcA. Both the PII-PipX complex structure and *in vivo* functional data suggested that this complex, as such, could have regulatory functions in addition to PipX sequestration. To investigate this possibility we performed yeast three-hybrid screening of genomic libraries from *Synechococcus elongatus* PCC7942, searching for proteins interacting simultaneously with PII and PipX. The only prey clone found in the search expressed PlmA, a member of the GntR family of transcriptional regulators proven here by gel filtration to be homodimeric. Interactions analyses further confirmed the simultaneous requirement of PII and PipX, and showed that the PlmA contacts involve PipX elements exposed in the PII-PipX complex, specifically the C-terminal helices and one residue of the tudor-like body. In contrast, PII appears not to interact directly with PlmA, possibly being needed indirectly, to induce an extended conformation of the C-terminal helices of PipX and for modulating the surface polarity at the PII-PipX boundary, two elements that appear crucial for PlmA binding. Attempts to inactive *plmA* confirmed that this gene is essential in *S. elongatus*. Western blot assays revealed that *S. elongatus* PlmA, irrespective of the nitrogen regime, is a relatively abundant transcriptional regulator, suggesting the existence of a large PlmA regulon. *In silico* studies showed that PlmA is universally and exclusively found in cyanobacteria. Based on interaction data, on the relative amounts of the proteins involved in PII-PipX-PlmA complexes, determined in western assays, and on the restrictions imposed by the symmetries of trimeric PII and dimeric PlmA molecules, a structural and regulatory model for PlmA function is discussed in the context of the cyanobacterial nitrogen interaction network.

## Introduction

**Figure 1:**
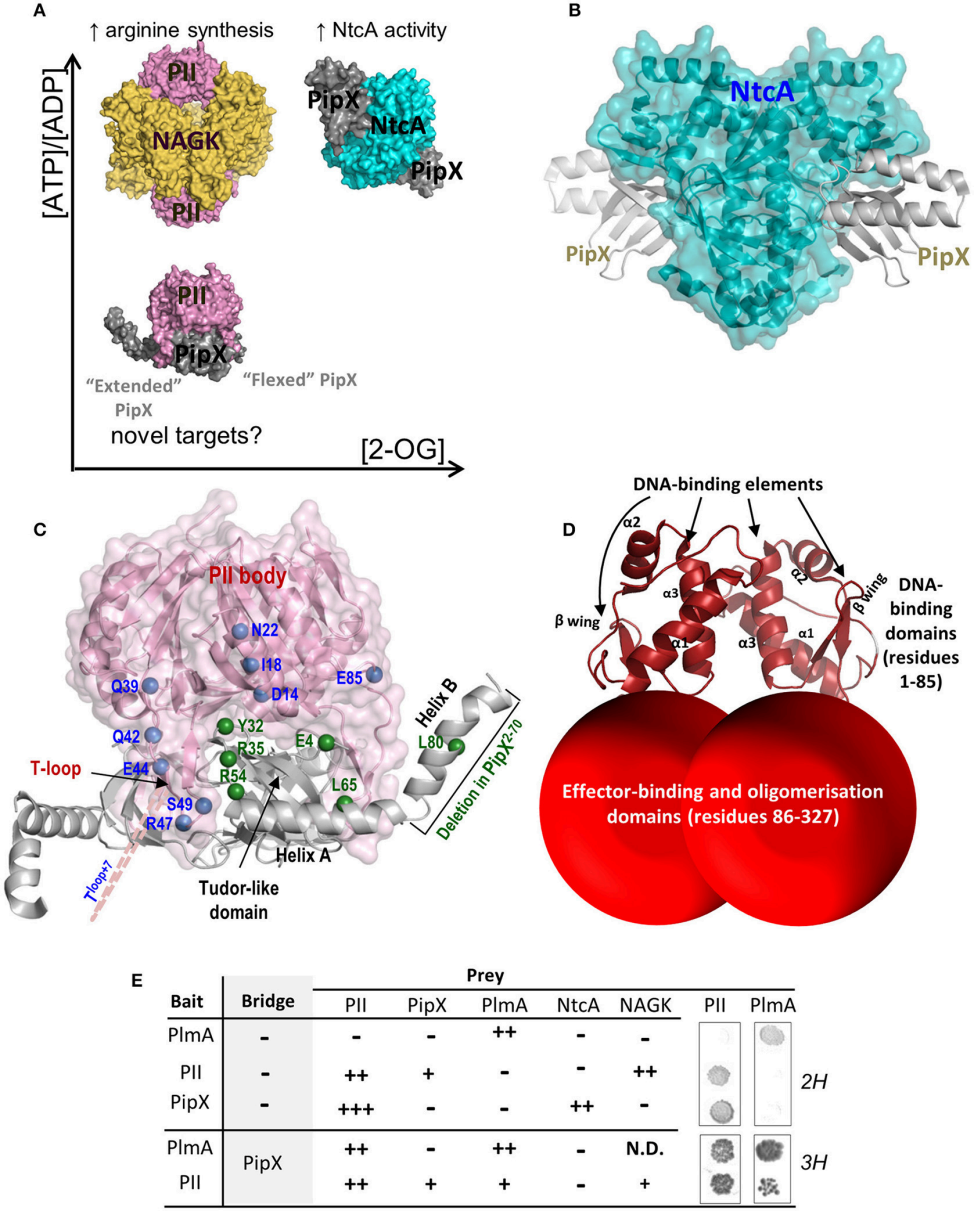
**Molecular players of nitrogen signaling and PlmA interactions**. **(A)** PII and PipX complexes formed according to the spectrum of 2-OG and ATP/ADP concentrations ratio, with indication of their known functions and of the possibility of previously unclear functions of the PII-PipX complex. **(B)** Structure of the NtcA-PipX complex of *S. elongatus*. The NtcA dimer is shown in semi-transparent surface representation to allow visualization of its secondary structure (in ribbons). The two bound PipX molecules are illustrated in ribbons representation and are colored gray. **(C)** PII-PipX complex of *S. elongatus*. The PII trimer is shown pink in semi-transparent surface representation with secondary structure visible below, with labeling of one T-loop and of the trimer body. PipX molecules are shown as ribbons (gray) with indication of the main structural elements of one of the three bound molecules. The residues mutated here are mapped in one PipX subunit (green spheres) having an “extended” C-terminal helix conformation and in PII (blue spheres) and are labeled. To show the position of mutated residues in relation to the PipX molecule, PII residues are marked in one of two adjacent PII subunits. The 7 residues insertion in the T-loop mutant is schematized with a dashed pink line. **(D)** Schematic model of the PlmA dimer. The N-terminal DNA binding winged helix-turn-helix (HTH) domains were modeled (http://swissmodel.expasy.org/) from the corresponding domains of the structure of DNA-bound YvoA (PDB file 4WWC; Fillenberg et al., 2015). The dimerization domain could not be modeled using currently available structures given the lack of an adequate template. The ratio of the volume of this last domain relative to the DNA-binding domain approximately corresponds to the relative chain lengths of both domains. **(E)** Yeast two-hybrid (2H) and three-hybrid (3H) interactions involving the indicated proteins. Symbols refer to the strength of interaction from no signal (−) to strong signals (+++). N.D, not determined. Representative examples of yeast diploids after 4 days of incubation on histidine deficient medium are shown at right.

Cyanobacteria are phototrophic organisms that perform oxygenic photosynthesis and require the assimilation of ammonia for autotrophic growth. This assimilation is carried out by the GS-GOGAT cycle, consuming 2-oxoglutarate (2-OG) (Muro-Pastor et al., 2001, 2005) which is an indicator of the carbon to nitrogen balance (Forchhammer, 2004; Laurent et al., 2005). 2-OG modulates the interactions of three key cyanobacterial proteins. Two of them, the signal transducer PII and the transcriptional regulator NtcA, bind 2-OG, whereas PipX binds to 2-OG-bound NtcA or to 2-OG-free PII (Figure 1A).

Studies in *Synechococcus elongatus* PCC7942 and *Anabaena* sp. PCC 7120 (hereafter *S. elongatus* and *Anabaena*, respectively) showed that activation of NtcA-dependent genes is enhanced by PipX binding to NtcA (Espinosa et al., 2006, 2007; Valladares et al., 2011; Forcada-Nadal et al., 2014). Under nitrogen limitation, 2-OG stimulates NtcA binding to target sites (Vázquez-Bermúdez et al., 2002), activating transcription (Tanigawa et al., 2002) and favoring complex formation between NtcA and PipX (Espinosa et al., 2006) (Figure 1A). The PipX-NtcA complex (Llácer et al., 2010; Zhao et al., 2010) consists of one NtcA dimer bound to 2 molecules of 2-OG and 2 of PipX (Figures 1A, B). PipX stabilizes the conformation of NtcA that is transcriptionally active and probably helps the local recruitment of RNA polymerase. Since the all-β N-terminal tudor-like domain of PipX provides the contacts of this protein with both NtcA (Figure 1B) and PII (Figure 1C), PII sequestration of PipX at low 2-OG renders PipX unavailable for NtcA binding and activation, reducing expression of NtcA-dependent gene targets (Figure 1A).

The homotrimeric PII protein, one of the most conserved and widespread signal transduction proteins, plays pivotal roles in nitrogen assimilatory processes (Leigh and Dodsworth, 2007). The PII trimer has three binding sites for ATP/ADP (and, in some species, AMP) and 2-OG (Fokina et al., 2010; Truan et al., 2010; Palanca et al., 2014). By making a complex with the key catalyst and controller of arginine biosynthesis, N-acetyl-L-glutamate kinase (NAGK) (Figure 1A), PII promotes nitrogen storage as arginine in cyanobacteria and plants (Burillo et al., 2004; Heinrich et al., 2004; Llácer et al., 2007). When abundant, 2-OG binds to MgATP-complexed PII, triggering conformational changes in the T-loops that prevent the interactions of PII with either NAGK or PipX (Espinosa et al., 2006; Llácer et al., 2010; Zeth et al., 2014). Complex formation with PipX increases the affinity of PII for ADP, and, conversely, the interaction between PII and PipX is highly sensitive to fluctuations in the ATP/ADP ratio (Zeth et al., 2014). Thus, PipX swapping between PII and NtcA links PII signaling with NtcA-regulated gene expression.

The structure of the PII-PipX complex shows that the two C-terminal helices of PipX (called here helices A and B) are accessible in the complex (Figure 1C), suggesting their possible involvement in additional regulatory interactions. Helix B, which is mobile and can be found in extended conformations projecting outwards, would provide an appropriate interacting element (Llácer et al., 2010). Novel interactions mediated by PII and PipX in cyanobacteria may provide a rationale for non-understood observations, including the need of PII for viability of *S. elongatus* at relatively low carbon to nitrogen rations (Chang et al., 2013) and the involvement of PipX in transcriptional regulation of cells grown in the presence of ammonium or nitrate (Espinosa et al., 2014). The formation of ternary complexes of PII with other proteins appears not to be exceptional, since PII complexes with the ammonium transporter AmtB and the transcriptional regulator TnrA or with such transporter and the nitrogenase regulatory enzyme DraG were reported, respectively, in *Bacillus subtilis* (Heinrich et al., 2006; Schumacher et al., 2015) and *Azospirillum brasilense* (Huergo et al., 2007).

In this work we searched for *S. elongatus* proteins interacting with PII-PipX complexes and identified PlmA, a poorly known regulator despite constituting one subfamily of the widely distributed GntR-like family (Hoskisson and Rigali, 2009), characterized by a conserved N-terminal winged helix-turn-helix (HTH) DNA-binding domain (Rigali et al., 2002; Zheng et al., 2009; Suvorova et al., 2015) and a diverse C-terminal dimerization/ligand-binding domain.

Functions in plasmid maintenance (Lee et al., 2003) and photosystem stoichiometry (Fujimori et al., 2005) have been proposed for the *Anabaena* and *Synechocystis* sp. PCC 6803 (hereafter *Synechocystis*) proteins, respectively. The two *plmA* mutants reported so far were identified in the context of genetic screenings for heterocyst development or altered chlorophyll fluorescent kinetics, suggesting that PlmA is a pleiotropic regulator controlling diverse biological processes.

We show here that PlmA does not interact with PII or PipX unless both proteins were co-expressed in the interaction assays. Insights into the significance of this finding were obtained by investigating (a) the specificity of the PII-PipX-PlmA interaction, (b) the molecular determinants of PII and PipX proteins involved in interactions with PlmA, (c) the quaternary structure of PlmA, (d) the importance of PlmA in *S. elongatus* (e) the *in vivo* levels of PlmA in relation to interaction partners PipX and PII and (f) the phylogenetic distribution and idiosyncrasy of PlmA.

## Materials and methods

### Biological reagents

The strains, plasmids and oligonucleotides used in this work are listed in Tables 1, 2. Rabbit antisera against PII and PipX proteins were donated by K. Forchhammer (Univ. Tübingen, Germany), whereas the one against PlmA was obtained from Pineda Antikörper Service (Berlin, Germany; http://www.pineda-abservice.de), using pure PlmA as antigen (details of PlmA preparation to be reported elsewhere). N-terminally His_6_-tagged PipX was a gift of JL Llácer (IBV-CSIC, Valencia) (Llácer et al., 2010). His_6_-tagged PII (sequence of the N-terminal tag, MH_6_SSGVDLGTENLYFQS) was produced in *E. coli* BL21 (DE3) cells transformed with pLIC-PII (see below), and it was purified as described for His_6_-tagged PipX, using Ni-affinity chromatography.

**Table 1:** **Strains and plasmids**.

**Strain or plasmid**	**Genotype or relevant characteristics**	**Source or references**
*Escherichia coli* DH5	F^−^φ80 d*lacZ*ΔM15Δ(*lacZYA-argF*)U169 *endA1 recA1 hsdR17*(${\text{r}}_{\text{K}}^{-}$ ${\text{m}}_{\text{K}}^{+}$) *deoR thi-1 supE44 gyrA96 relA1*λ^−^	Hanahan, 1985
*Escherichia coli* HB101	F^−^*mcr*B *mrr hsd*S20(${\text{r}}_{B}^{-}$ ${\text{m}}_{B}^{-}$) *rec*A13 *leu*B6 *ara*-14 *pro*A2 *lac*Y1 *gal*K2 *xyl*-5 *mtl*-1 *rps*L20(Sm^R^) *gln*V44λ^−^	Hanahan, 1985
*Escherichia coli* Bl21 (DE3)	*huA2 [lon] ompT gal (λ DE3) [dcm] ΔhsdS λ DE3 = λ sBamHIo ΔEcoRI-B int::(lacI::PlacUV5::T7 gene1) i21 Δnin5*	Novagen
*Saccharomyces cerevisiae* Y187	MAT*α ura3-52 his3-200 ade2-101 trp1-901 leu2-3, 112 gal4Δmet^−^ gal80Δ URA::GAL1_*UAS*_-GAL1_*TATA*_-lacZ*	Harper et al., 1993
*Saccharomyces cerevisiae* PJ696	MATα *ade*2Δ*trp*1-901 *leu*2-3,112 *ura*3-52 *his*3-200 *cyh*^R^ *can*^R^ *gal*4Δ*gal*80Δ *met*2^−^*GAL*2::*ADE2 GAL1*::*HIS3* GAL7:*lacZ*	James et al., 1996
WT	*Synechococcus elongatus* wild-type	Pasteur culture collection
1Ptrc	WT carrying Ptrc promoter into NSI, Sm^R^	Moronta-Barrios et al., 2013
1Ptrc-PlmA	Φ(P*trc*::*plmA*) NSI, Sm^R^	This work
1Ptrc-PlmA PCK1	Φ(P*trc*::*plmA*) NSI, *plmA::*C.K1-, Sm^R^ Km^R^	This work
pBridge	*TRP1*, MCSI GAL4(1-147) BD, MCSII	Clontech
pGAD424	Amp^R^, *LEU2*, GAL4(768-881) AD	Bartel et al., 1993
pGBT9	Amp^R^, *TRP1*, GAL4(1-147) BD	Bartel et al., 1993
pGAD424(+2)	As pGAD424 with a different frame (+2)	Roder et al., 1996
pGBT9(+2)	As pGBT9 with a different frame (+2)	Roder et al., 1996
pBluescript SK+	Cloning vector	Stratagene
pLIC-SGC1	pET expression vector, His_6_, Ap^R^	Gileadi et al., 2008
pLIC-PII	His-tagged *glnB*, His_6_-PII, Ap^R^	This work
pUAGB001	GAL4BD, HA: PipX, Ap^R^	This work
pUAGB111	GAL4BD:PII, HA:PipX, Ap^R^	This work
pUAGC854	GAL4BD:PlmA, HA:PipX, Ap^R^	This work
pUAGC858	GAL4BD:PlmA, HA:PipX^2−70^, Ap^R^	This work
pUAGC705	GAL4AD:PipX^Y32A^, Ap^R^	Llácer et al., 2010
pUAGC487	GAL4AD:PipX^R35A^, Ap^R^	Llácer et al., 2010
pUAGC391	Allele C.S3-*pipX^R54C^*, Sm^R^ Ap^R^	Espinosa et al., 2010
pUAGC390	Allele C.S3-*pipX^L65Q^*, Sm^R^ Ap^R^	Espinosa et al., 2010
pUAGC474	GAL4BD:PipX^E4A^, Ap^R^	Llácer et al., 2010
pUAGC706	GAL4BD:PipX^Y32A^, Ap^R^	Llácer et al., 2010
pUAGC488	GAL4BD:PipX^R35A^, Ap^R^	Llácer et al., 2010
pUAGC498	GAL4BD:PipX^R54C^, Ap^R^	Llácer et al., 2010
pUAGC372	GAL4BD:PipX^L65Q^, Ap^R^	Llácer et al., 2010
pUAGC207	GAL4BD:PlmA, HA:PipX^E4A^, Ap^R^	This work
pUAGC206	GAL4BD:PlmA, HA:PipX^Y32A^, Ap^R^	This work
pUAGC198	GAL4BD:PlmA, HA:PipX^R35A^, Ap^R^	This work
pUAGC197	GAL4BD:PlmA, HA:PipX^L65Q^, Ap^R^	This work
pUAGC199	GAL4BD:PlmA, HA:PipX^R54C^, Ap^R^	This work
pUAGC208	GAL4BD:PlmA, HA:PipX^L80Q^, Ap^R^	This work
pUAGC831	GAL4AD:PlmA, Ap^R^	This work
pUAGC832	GAL4BD:PlmA, Ap^R^	This work
pUAGC709	GAL4AD:PipX^2−70^, Ap^R^	This work
pUAGC710	GAL4BD:PipX^2−70^, Ap^R^	This work
pUAGC703	GAL4AD:PipX^L80Q^, Ap^R^	This work
pUAGC704	GAL4BD:PipX^L80Q^, Ap^R^	This work
pUAGC6	GAL4AD:NtcA, Ap^R^	Espinosa et al., 2010
pUAGC8	GAL4BD:NtcA, Ap^R^	Espinosa et al., 2010
pUAGC11	GAL4AD:PII, Ap^R^	Burillo et al., 2004
pUAGC12	GAL4BD:PII, Ap^R^	Burillo et al., 2004
pUAGC13	GAL4AD:PII^S49A^, Ap^R^	Burillo et al., 2004
pUAGC15	GAL4AD:PII^S49D^, Ap^R^	Burillo et al., 2004
pUAGC17	GAL4AD:PII^S49E^, Ap^R^	Burillo et al., 2004
pUAGC529	GAL4AD:PII^D14A^, Ap^R^	Llácer et al., 2007
pUAGC531	GAL4AD:PII^I18A^, Ap^R^	Llácer et al., 2007
pUAGC533	GAL4AD:PII^N22A^, Ap^R^	Llácer et al., 2007
pUAGC537	GAL4AD:PII^Q39A^, Ap^R^	Llácer et al., 2007
pUAGC539	GAL4AD:PII^Q42A^, Ap^R^	Llácer et al., 2007
pUAGC523	GAL4AD:PII^E44A^, Ap^R^	Llácer et al., 2007
pUAGC543	GAL4AD:PII^R47A^, Ap^*R*^	Llácer et al., 2007
pUAGC521	GAL4AD:PII^E85A^, Ap^R^	Llácer et al., 2007
pUAGC471	GAL4AD:PipX, Ap^R^	Espinosa et al., 2010
pUAGC472	GAL4BD:PipX, Ap^R^	Espinosa et al., 2010
pUAGC61	GAL4AD:NAGK, Ap^R^	Burillo et al., 2004
pUAGC62	GAL4BD:NAGK, Ap^R^	Burillo et al., 2004
pUAGC373	GAL4AD:PII^T−loop+7^, Ap^R^	Espinosa et al., 2010
pUAGC374	GAL4BD:PII^T−loop+7^, Ap^R^	Espinosa et al., 2010
pRL161	C.K1 Km^R^ cartridge	Elhai and Wolk, 1988
pUAGC453	C.S3 into pBluescript SK+	Ruiz et al., 2008
pUAGC833	*plmA* region into pBluescript SK+	This work
pUAGC836	*plmA::*C.K1 into *plmA*, Km^R^, Ap^R^	This work
pUAGC837	*plmA::*C.K1 into *plmA*, Km^R^, Ap^R^	This work
pUAGC835	*plmA::*C.S3 into *plmA*, Sm^R^, Ap^R^	This work
pUAGC839	Ptrc::*plmA* allele into NSI, Sm^R^, Ap^R^	This work
pUAGC280	Ptrc promoter into NSI, Sm^R^, Ap^R^	Moronta-Barrios et al., 2012

**Table 2:** **Oligonucleotides**.

**Name**	**Sequence**
ACTAseq	5′ AGGGATGTTTAATACCACTAC 3′
GAD-REV	5′ GTTGAAGTGAACTTGCGG 3′
Transgadgbt-1F	5′CGCACATCATCATCGGAAGAGAGTAGTAACAAAGGTCAAAGACAGTTGACTGTATCGCCGAACCCAAAAAAAGAGATCG 3′
Transgadgbt-1R	5′ATAACTTATTTAATAATAAAAATCATAAATCATAAGAAATTCGCCCGGAATTAGCTTGGCGTTTTTCAGTATCTACGATTC 3′
PipX pBridge 1F	5′ GATTCCCCGCGGCCGCGGCTTCCGAG 3′
PipX pBridge 1R	5′ GCAGATCTCTACAGAAAGGTTTGTTTG 3′
PipX-L80Q-F	5′ GCAGGAATACAACCAGCAGCAGCAAGTCTTCAAAC 3′
PipX-L80Q-R	5′ GTTTGAAGACTTGCTGCTGCTGGTTGTATTCCTGC 3′
PipX-pBridge-E4A-F	5′ GCGGCCGCGGCTTCCGCGAACTACCTC 3′
plmA-YTH-1F	5′ GGTTCGAATTCAATGATTCGTTTTCAC 3′
plmA-YTH-1R	5′ ACACCGGATCCTGTGGTTTAGTTCAAACC 3′
PipX-OV-2F	5′ GAGAATTCGCTTCCGAGAACTACC 3′
PipXresi70Rev	5′ GCAGGAATTCCTATCGGCGCAGCTGGCGC 3′
plmA-inact-1F	5′ GCCACGAATTCGCCCACGACAGG 3′
plmA-inact-1R	5′ TAATCCTCGAGGTGTTTTCGCCG 3′
pMAL-plmA-1F	5′ ATCGGAATTCATGATTCGTTTTCACATCC 3′
pMAL-plmA-1R	5′ ATCGGGATCCTTAGTTCAAACCCAGTTCCC 3′
pLIC-PII-F	5′ TACTTCCAATCCATGAAGAAGATTGAGGCG 3′
pLIC-PII-R	5′ TATCAACCTTTACTGTTAGATTGCGTCGGC 3′
qPCR-plmA-1F	5′ GATCAATCCAGCATTGACAA 3′
Sip1-BTH-F	5′ GGGGGTACCTTGATTCAGAC 3′
Sip1-BTH-R	5′ GATCGGGATCCCCGAGTAATG 3′
PTRC99Aseq-F	5′ GCCGACATCATAACGG 3′
NSI-1F	5′ CGACATCTTCCTGCTCCAG 3′

### Molecular genetic techniques and growth conditions

Cloning procedures were carried out with *Escherichia coli* DH5α, using standard techniques (Sambrook et al., 1989). Constructs and mutations were analyzed by automated dideoxy DNA sequencing. Yeast culture and transformation procedures were as described (Ausubel et al., 1999). *S. elongatus* strains were routinely grown photoautotrophically at 30°C while shaking under constant illumination (40 μmol photons m^−2^ s^−1^) provided by cool white fluorescent lights. Media used were BG11_0_ (lacking the nitrogen source), BG11 (BG11_0_ supplemented with 17.5 mM NaNO_3_) or BG11^A^ (BG11_0_ supplemented with 5 mM NH_4_Cl and 10 mM HEPES/NaOH pH 7.8). For growth on plates, the media was solidified by addition of 1% (w/v) agar. Plates were routinely incubated at 30°C under constant illumination. *S. elongatus* strains were transformed essentially as described (Golden and Sherman, 1984). Whenever used, antibiotic concentrations for *S. elongatus* were 10 μg kanamycin ml^−1^ and 5 μg streptomycin ml^−1^.

### Yeast two and three-hybrid assays

To perform yeast three-hybrid screenings, previously obtained *S. elongatus Sau3*AI libraries (Burillo et al., 2004) were transformed into *Saccharomyces cerevisiae* PJ696 and mated with strain Y187 carrying pUAGB111 which expresses BD:PII and PipX as a bridge protein. Transconjugants were selected on YNB minimal medium lacking Leu, Trp and His and containing 5 mg l^−1^ of adenine (Ade). Candidate clones were transferred to fresh medium and their library plasmid (prey plasmid) isolated from yeast and used to transform electrocompetent *E. coli* HB101. Prey plasmids were rescued, sequenced and retransformed into PJ696. To confirm the specificity of the interactions, matings were performed with yeast strain Y187 carrying bait or vector plasmids. To determine interaction patterns among selected proteins, expression from the reporters *HIS3* and *ADE2* in PJ696/Y187 diploids was summarized as previously described (Burillo et al., 2004). To improve detection of weak interaction signals we carried out a high sensitivity assay: Saturated liquid cultures of PJ696/Y187 diploids in control media (YNB + Ade + His) were centrifuged and concentrated 10 times before 5 μl were spotted onto selective media plates (YNB lacking His or Ade).

### Plasmids construction

To construct plasmids pUAGC831 and pUAGC832, the *plmA* coding sequence (*Synpcc7942_0090* Cyanobase gene identifier) was amplified from the *S. elongatus* genome with primers plmA-YTH-1F and plmA-YTH-1R and cloned into the *EcoR*I-*Bam*HI sites of pGAD424 (+2) and pGBT9 (+2), respectively. The *pipX* coding sequence was amplified from *S. elongatus* genomic DNA with primers PipX pBridge 1F and PipX pBridge 1R and cloned into the *Not*I-*Bgl*II site of pBridge (Clontech), giving plasmid pUAGB001. A *Xho*I-*Bam*HI fragment containing *plmA* sequences from pUAGC832 was cloned into pUAGB001, giving plasmid pUAGC854. *glnB* coding sequences were amplified from pUAGC11 with primers Transgadgbt-1F and Transgadgbt-1R. The PCR product was co-transformed with pUAGB001 (cut with *Eco*RI and *Sal*I) into yeast strain Y187, giving plasmid pUAGB111. Sequences coding for PipX^Y32A^, PipX^R35A^, PipX^R54C^, and PipX^L65Q^ were PCR amplified from pUAGC705, pUAGC487, pUAGC391, and pUAGC390, respectively, with primers PipX pBridge 1F and PipX pBridge 1R. The resulting PCR products were co-transformed with pUAGC854 (*Bpu*1102I and *Psh*AI digested) into Y187, giving plasmids pUAGC206, pUAGC198, pUAGC199 and pUAGC197. The sequence coding for PipX^E4A^ was amplified from pUAGC471 with primers PipX-pBridge-E4A-F and PipX pBridge 1R, and cloned into *Not*I and *Blg*II-digested pUAGC854, giving plasmid pUAGC207. QuickChange Mutagenesis with primers PipX-L80Q-F and PipX-L80Q-R and plasmids pUAGC471, pUAGC472, and pUAGC854 as templates resulted in plasmids pUAGC703, pUAGC704, and pUAGC208, respectively. To obtain PipX^2−70^, the *pipX* sequence encoding amino acids 2–70 was amplified using PipX-OV-2F and PipXresi70Rev and cloned into *Eco*RI-digested pGAD424 and pGBT9 vectors, giving plasmids pUAGC709 and pUAGC710, respectively. To generate plasmid pUAGC858, *pipX* sequences were amplified from pUAGC709 using primers ACTAseq and GAD-rev, cut with *Blp*I and *Sma*I and cloned into *Blp*I and *PshA*I-digested pUAGC854.

To generate pLIC-PII, expressing recombinant His-tagged PII, *S. elongatus glnB* sequences obtained by amplification with primers pLIC-PII-F and pLIC-PII-R were cloned into pLIC-SGC1 (Gileadi et al., 2008).

To inactivate *plmA*, first a 1.1 Kb DNA fragment was amplified from the *S. elongatus* genome using primers plmA-inact-1F and plmA-inact-1R and cloned into *Eco*RI and *Xho*I digested pBluescriptSK+, yielding plasmid pUAGC833. A *Hinc*II fragment from pRL161, containing a kanamycin (Km) resistant cassette, or a *Hinc*II-*Eco*RV fragment from pUAGC453, containing a streptomycin (Sm) cassette, were cloned into *Hinc*II and *Eco*RV digested pUAGC833. The resulting plasmids carry the *plmA* coding sequence interrupted by genes conferring resistance to Km (pUAGC836 and pUAGC837) or Sm (pUAGC835).

To overexpress PlmA, its ORF was PCR amplified with pMAL-plmA-1F and pMAL-plmA-1R, digested with *Eco*RI and *Bam*HI and cloned into pUAGC280. The resulting plasmid, named pUAGC839, carries *plmA* under control of the IPTG-inducible promoter Ptrc.

### Immunodetection of PII, PipX, and PlmA in *S. elongatus*

*S. elongatus* wild type or mutant strains (*pipX* and *pipX glnB*) were grown in BG11^A^ media to an OD_750_ of approximately 0.5 (mid-exponential growth phase). To determine protein amounts in ammonia-growing cells, 200 mL cultures were collected by centrifugation (8000 × g, 5 min 4°C). Cell pellets were quickly frozen in liquid nitrogen and stored at −80°C until use. Further operations were at 4°C. The cells, tenfold-diluted in 10 mM Tris-HCl pH 7.5, 1 mM β-mercaptoethanol, 0.5 mM EDTA, and 1 mM phenyl methyl sulphonyl fluoride (PMSF) were vortexed 5 min with glass beads. The decanted bead-free fluid was mixed with an excess of SDS-PAGE sample buffer, heated 5 min in a boiling water bath, and subjected to SDS-PAGE in 15% polyacrylamide gels, followed by western blotting to nitrocellulose membranes and immunoblotting with an appropriate antiserum (Forchhammer and Tandeau de Marsac, 1995). The amount of total protein in the extract applied per well was 20 μg. Measured amounts of purified recombinant His-tagged PII, His-tagged PipX or PlmA were run in parallel in the same gels. For PII, PipX, and PlmA immunodetections the corresponding antisera were diluted to 1:5000–1:7500 in 50 mM Tris-HCl pH 7.5 containing 200 mM NaCl (TBS). For visualization, a horseradish peroxidase-conjugated anti-rabbit IgG (Promega) at 1:5000 dilution was used, with luminescent detection with the ECL reagents and an ImageQuant LAS 4000 Mini biomolecular imager (both from GE Healthcare). Quantification was performed using the Multi Gauge V2.1 program from FUJIFILM. To determine PlmA protein levels and PII phosphorylation state in cells growing under different nitrogen conditions, ammonium-growing cells were collected by centrifugation at ambient temperature and washed twice with BG11_0_ medium to remove the remaining ammonia. Washed cells were used to inoculate fresh media supplemented with ammonium, nitrate or lacking nitrogen, and were incubated for another 2 h prior to cell pelleting by centrifugation at 4°C and immediate freezing of the cells until use. Cell pellets from 10 ml of culture were lysed as above, centrifuged, and the supernatants were subjected to SDS-PAGE (5–20% polyacrylamide linear gradient) or to 10% SDS-PAGE using Phos-tag technology (Wako, Japan), followed by immunoblotting as above except for the use of 0.1 μm PVDF membranes (from GE Healthcare), the utilization of 1:10^4^ dilutions of the primary anti-PlmA or anti-PII antisera, and the use of the secondary anti-rabbit-HRP antibody at 1:10^5^ dilution. Visualization was performed with SuperSignal WestFemto reagent (from Pierce) followed by auto-radiographic detection on X-ray films.

To carry out the analysis of *plmA* expression levels in *S. elongatus*, RT-PCR assays were performed using 50 ng of DNase-treated total RNA, isolated as described (López-Redondo et al., 2010). After retrotranscription of *plmA* and *sipA* mRNAs using the plmA-YTH-1R and Sip1-BTH-R primers, respectively, cDNA (90 ng) was used as template in a 25-cycle PCR reaction with primers qPCR-plmA-1F/plmA-YTH-1R for *plmA* and Sip1-BTH-F/R for *sipA*. 10 μL of the PCR reactions were loaded in a 1.5% agarose gel to visualize the amplified products.

### Computational methods

Homemade bidirectional blast analyses using the Refseq genomic bacterial database (http://www.ncbi.nlm.nih.gov/refseq) and *S. elongatus* PlmA and NtcA amino acid sequences as queries, were used to obtain homologous sequences. Each homolog was blasted against the annotated proteome of *S. elongatus* and if the first hit obtained was the original query (PlmA or NtcA) it was annotated as a real ortholog, otherwise discarded. A total of 102,738 and 33,903 hits were obtained for PlmA and NtcA, respectively. Orthologs of GntR-subfamilies (only PlmA, DevA and MocR orthologs are shown in **Figure 5**) were obtained using the same blast approach. To confirm the hits as members of one GntR subfamiliy each one was blasted against a database containing one representative protein sequence of each GntR subfamily: DevA (accession ), MocR (accession P49309), PlmA (accession ABB56122), AraR (accession P96711), FadR (accession P0A8V6), HutC (accession P22773), and YtrA (accession O34712). The neighbor-joining phylogenetic tree for PlmA orthologs (234 sequences from bidirectional blast search) was constructed using the web tool ClustalW (Sievers et al., 2011) with default parameters, and visualized with iTOL (Letunic and Bork, 2016).

### Other methods

Protein was determined (Bradford, 1976) with a commercial dye reagent (from Bio-Rad) using bovine serum albumin as standard. However, with *S. elongatus* extracts, to avoid chlorophyll interference, protein was determined according to Lowry (Lowry et al., 1951), again with a commercial Lowry reagent (Modified Lowry protein Assay Kit, from Thermo Scientific) and bovine serum albumin as standard, measuring the absorbance at 750 nm.

Protein structures were represented using PyMOL 0.99rc6 (DeLano Scientific LLC), utilizing the Protein DataBank (PDB; http://www.rcsb.org/pdb/) files 2XG8, 2XKO, and 2V5H for PII-PipX, NtcA-PipX, and PII-NAGK, respectively (Llácer et al., 2007, 2010).

## Results and discussion

### Rationale to search for proteins interacting with PII-PipX complexes

Yeast two-hybrid approaches allowed significant contributions to our understanding of the cyanobacterial nitrogen regulatory network. In particular, yeast two-hybrid screenings of *S. elongatus* libraries with PII as bait identified NAGK and PipX as preys (Burillo et al., 2004; Heinrich et al., 2004). Subsequent characterization of PII-NAGK, PII-PipX and NtcA-PipX complexes in *S. elongatus* and other organisms also benefited from two-hybrid approaches (Llácer et al., 2007, 2010; Zhao et al., 2010).

We reasoned that since the PII-PipX complex is formed at relatively low ratios of carbon/nitrogen and ATP/ADP (Figure 1A), it might be signaling these metabolic conditions to proteins in charge of eliciting appropriate transcriptional responses. If this was the case, binding of the hypothetical protein(s) to the PII-PipX complex would probably involve complex-specific surfaces absent when the proteins are considered separately, or as part of other complexes such as PII-NAGK or PipX-NtcA.

The yeast three-hybrid system (Brachmann and Boeke, 1997; Tirode et al., 1997), allowing detection of interactions between two proteins (bait and prey) in the presence of a third one (bridge), was the method chosen to search for prey proteins interacting with PII-PipX complexes.

### A three-hybrid search for proteins interacting with PII-PipX complexes identified the GntR-like regulator PlmA

A bait plasmid expressing GAL4BD-PII (bait fusion) and PipX (bridge protein), was constructed and subsequently used to screen genomic libraries containing *S. elongatus Sau3A*I fragments fused to GAL4AD coding sequences (prey fusions). Three-hybrid screenings produced, in addition to clones containing *glnB* sequences found in our previous two-hybrid screenings with either GAL4BD-PII or GAL4BD-PipX as baits (Burillo et al., 2004 and data not shown), non-anticipated clones containing *Synpcc7942_0090* (Cyanobase Gene Identifier http://genome.microbedb.jp/cyanobase/SYNPCC7942) sequences. These identified the ortholog of the *Anabaena* GntR-like putative transcriptional regulator PlmA, for which a function in plasmid maintenance has been proposed (Lee et al., 2003). PlmA belongs to the widely distributed GntR-family of transcriptional regulators (Hoskisson and Rigali, 2009), characterized by a conserved N-terminal winged helix-turn-helix (HTH) DNA-binding domain (Rigali et al., 2002; Zheng et al., 2009; Suvorova et al., 2015) and a diverse C-terminal dimerization/ligand-binding domain. Based on this information, a model for PlmA structure in shown in Figure 1D.

To validate and gain further insights into the interaction detected between PII, PipX, and PlmA proteins, yeast two-hybrid and three-hybrid assays were performed with different combinations of these and additional proteins from the nitrogen interaction network used as controls (NtcA and NAGK). To this end, we constructed full-length derivatives of these proteins fused to GAL4AD or GAL4BD domains. For three-hybrid “bait plasmids,” in addition to GAL4BD-PlmA or GAL4BD-PII fusions, PipX was also expressed as the bridge protein. Results from two- and three-hybrid analyses with full length proteins are summarized in Figure 1E.

**Figure 2:**
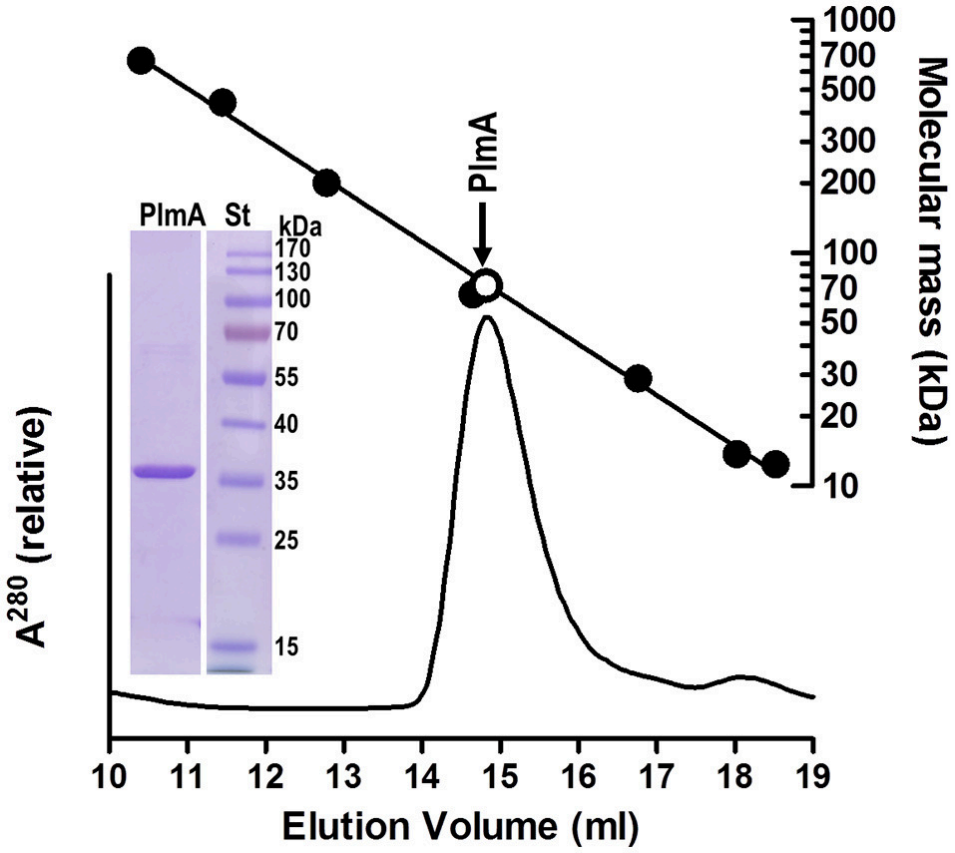
**Size exclusion chromatography of PlmA indicates that it is a dimer**. Pure recombinant PlmA (0.1 mg; details of production to be reported elsewhere; purity illustrated in the inset showing Coomassie-stained 12% SDS-PAGE gel; St, PageRuler prestained mass standards, from Thermo Scientific) was applied in 0.1 ml to a SuperdexTM 200 10/300 GL column (GE Healthcare) mounted on a ÄKTA FPLC system run at 0.3 ml/min of 50 mM Hepes, pH 7.5, 0.5 M NaCl, and 1 mM 2-mercaptoethanol, monitoring the optical absorption of the effluent at 280 nm (bottom graph). A semilogarithmic plot of mass relative to elution volume of protein standards is shown at the top. This plot was prepared with thyroglobulin, ferritin, β-amylase, bovine serum albumin, carbonic anhydrase, ribonuclease A, and cytochrome C, having respective masses (in kDa) of 669, 443, 200, 66.4, 29, 13.7, and 12.4. The open circle marks the elution position of the PlmA peak assuming that it is a dimer (sequence-deduced mass for the dimer, 72.7 kDa).

When tested against other fusion proteins in two-hybrid assays, PlmA constructs gave interaction signals with itself (PlmA-PlmA) but not with PII, PipX, NtcA, or NAGK, confirming that PlmA does not interact with PipX or PII unless the third protein is present in the yeast assays and further suggesting a homo-oligomeric structure for PlmA. In this context, most known GntR regulators are dimers (Rigali et al., 2002) although tetrameric structures have also been proposed (Rigali et al., 2002; Kataoka et al., 2008; Okuda et al., 2015). A homodimeric organization (modeled in Figure 1D) was supported by gel filtration experiments with purified PlmA (Figure 2). Consistent with the strong interaction signals when PII or PlmA are paired with themselves in yeast assays, expression of PipX had negligible effects on PII-PII and PlmA-PlmA interactions. The results from two- and three-hybrid assays confirmed the specificity of the novel interaction between PlmA and the two proteins PII and PipX when co-expressed in yeast. In addition, the results indicated that the combination GAL4AD-PII, GAL4BD-PlmA and PipX as bridge gave stronger interaction signals than the one obtained from library searches (GAL4AD-PlmA, GAL4BD-PII, and PipX as bridge), prompting us to use it as the starting point for subsequent analyses with mutant variants.

### Mutational analyses support the requirement of PII-PipX complex formation and the involvement of the A and B helices of PipX for interaction with PlmA

If, as inferred from the above results, PlmA interacts with PipX/PII when these two proteins are in complex, we can anticipate two types of mutations impairing three-hybrid interactions: Those that impair formation of the PII-PipX complex and those targeting residues involved in direct contacts with PlmA. With this in mind, subsequent yeast three-hybrid analyses were carried out with mutant derivatives of PipX or PII.

**Figure 3:**
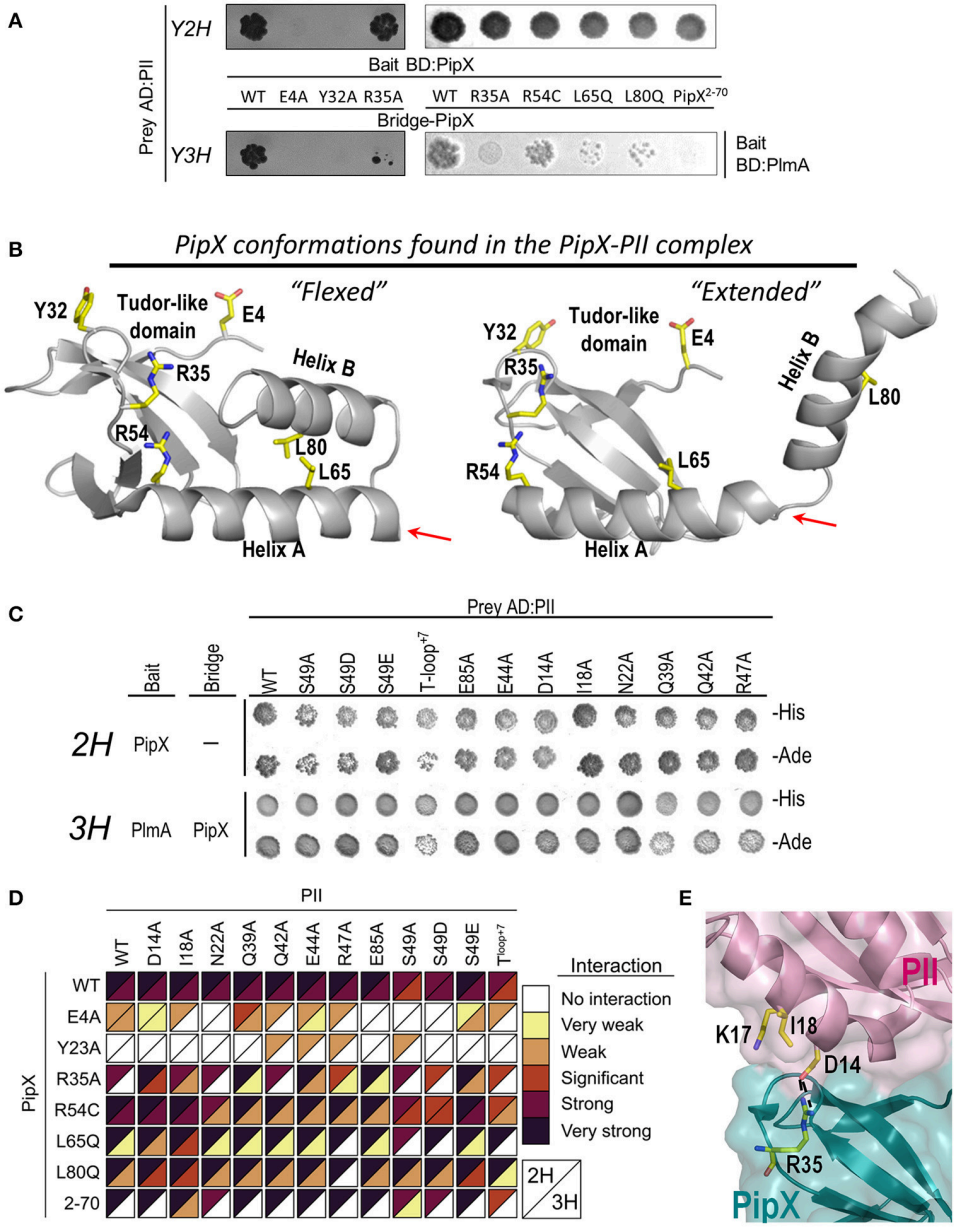
**Effects of PipX and PII mutations on two-hybrid and three-hybrid interaction assays**. **(A)** Growth conferred by the indicated PipX derivatives in comparative yeast 2-hybrid (2H) and 3-hybrid (3H) assays on histidine deficient media. **(B)** Localization of the PipX residues mutated here on the structures of the “flexed” and “extended” conformations of this protein as observed in the PII-PipX complex of *S. elongatus* (taken from PDB file 2XG8) (Llácer et al., 2010). These residues are shown with their side-chains in yellow (O and N atoms red and blue, respectively) and are labeled. The right arrow marks the point of truncation in PipX^2−70^. **(C)** Effects of PII mutations in 2H and 3H assays. Pictures were taken after 4 days of incubation on histidine (-His) or adenine (-Ade) deficient media. **(D)** Heatmap summarizing results from 2H and 3H assays involving PII and PipX mutant derivatives. The color scale refers to the strength of the interaction signals in standard (from significant to strong interaction) and high sensitivity (from no to weak interaction) assays (see Methods). **(E)** Detail of PII-PipX interactions around R35 of PipX in the structure of the PII-PipX complex of *S. elongatus* (PDB 2XG8) to illustrate the effects of the PII mutations D14A and I18A.

To get insights into the PlmA/PipX/PII interactions we first analyzed PipX^E4A^, PipX^Y32A^, and PipX^R35A^ fusion proteins, which affect interactions with PII to different extents (Llácer et al., 2010; Laichoubi et al., 2012). Mutations E4A and Y32A completely abolish interactions with PII in two-hybrid assays, in contrast to the minor impact of mutation R35A. As shown in Figures 3A,D, all three mutations resulted in lack of interaction signals in three-hybrid assays. While results with PipX^E4A^ and PipX^Y32A^ fusion proteins supported the importance of previous formation of the PipX-PII complex for contacts with PlmA, those with PipX^R35A^ suggest that Arg35 plays a role in connecting PipX with PlmA.

The C-terminal helices of PipX appear privileged candidates for interaction with PlmA, since they are observed in the PII-PipX complex in “extended” conformations, with helix B protruding and being widely exposed while the outwards-looking face of helix A also becomes exposed (Figures 1A,C, 3B) (Llácer et al., 2010). To test the importance of these C-terminal helices in the interaction with PlmA, we compared the behavior on two- and three-hybrid assays of PipX^R54C^, PipX^L65Q^, PipX^L80Q^, and PipX^2−70^ fusion proteins. The R54C and L65Q changes, not impairing two-hybrid interactions with PII, were originally found as spontaneous suppressor mutations from cultures where *glnB* was previously inactivated and are thus predicted to impair relevant *in vivo* functions of PipX (Espinosa et al., 2009). PipX^2−70^ and PipX^L80Q^ fusion proteins target helix B, by deleting it or introducing a point mutation, respectively. L80 was chosen for substitution because it interacts with L65 in the “flexed” conformation of PipX, while, similarly to L65, it is exposed in the “extended” conformation and could be a good candidate for interaction with PlmA (Figure 3B).

As expected (Espinosa et al., 2009; Llácer et al., 2010), two-hybrid interactions with PII were not perturbed by PipX^R54C^, PipX^L65Q^, PipX^L80Q^, and PipX^2−70^ fusion proteins (Figure 3A). While three-hybrid interaction signals with PlmA were not significantly affected with PipX^R54C^, they were strongly impaired with PipX^L65Q^ and PipX^L80Q^ and abolished with PipX^2−70^ (Figure 3A). These results strongly support the importance of the C-terminal helices and reveal that helix B is essential for interaction with PlmA.

The important impairment on three-hybrid signals produced by PipX^L65Q^ or PipX^L80Q^ is best reconciled with a model in which PlmA binds to the “extended” conformation of PipX, with the exposed residues L65 and L80 directly involved in the interactions with PlmA, possibly mediating these interactions by hydrophobic contacts. On the other hand, R54 is away from the center of helix A and its highly polar side-chain is exposed in both the “flexed” and the “extended” PipX conformations (Figure 3B). The irrelevance of R54 for three-hybrid interactions argues against its participation in PlmA binding.

### PII may not establish direct protein-protein contacts with PlmA

The above discussed three-hybrid results indicated the unambiguous involvement of the C-terminal helices of PipX as well as the need for PII in order to detect interactions signals, but did not inform on the role of PII. In particular, whether PII also binds PlmA or just provides the “extended” conformation of PipX helices which is apparently required for contacts. To distinguish between these two scenarios, we performed additional assays with PII variants whose mutations did not impair PII-PipX interactions (Llácer et al., 2007; Espinosa et al., 2009) but did affect residues at the surface of PII-PipX complexes close to PipX (Figure 1C). They target five residues of the T-loop (the loop that entraps PipX; mutations introduced, Q39A, Q42A, E44A, R47A, S49A/E/D; S49 is the phosphorylatable residue), three at the first helix of PII (D14A, I18A, and N22A), one in the B-loop (E85A) and also a 7-amino acids insertion after E44 in the T-loop (PII^T−loop+7^; spontaneous mutation, Espinosa et al., 2009).

None of the tested PII mutations resulted in a significant decrease of three-hybrid interaction signals (Figure 3C and first row of Figure 3D), arguing against the direct involvement of PII in interactions with PlmA and in favor of a role on providing the “extended” PipX conformation. It should be noted that this proposed role of PII in holding PipX in the right conformation to bind to PlmA could not be fulfilled by NtcA, due to the different conformation adopted by PipX in complex with NtcA (Figure 1B) (Llácer et al., 2010). In line with this, when NtcA instead of PII is used as prey, no three-hybrid interaction signals are detected with PlmA (Figure 1E).

### Mutational analyses support the importance of surface potential on PII/PipX/PlmA interactions

To get additional insight into the role of PII and PipX on the contacts with PlmA, we wondered whether some of the PII mutations discussed in this work could compensate the negative effects on three-hybrid interactions of the PipX mutations tested here. With this in mind, two- and three-hybrid assays were performed for all pair combinations of wild type and mutant derivatives of PipX and PII. To integrate information from multiple assays and facilitate comparisons between the different proteins and controls included, an illustrative figure summarizing results of two-hybrid and three-hybrid analyses in a semi-quantitative scale (from “no” to “very strong” interaction signals according to color intensities of triangles for each type of assay) is provided in Figure 3D. Note that this table also includes results that have been previously illustrated, with representative examples, in Figures 3A,C.

Some of the paired fusion proteins did result in compensatory effects, increasing only three-hybrid interaction signals or doing so to a greater extent than on two-hybrid assays. The more prominent effect in this context was observed for the pair PII^D14A^/PipX^R35A^, affecting two residues that form an ion pair in the surface of the PII-PipX complex (Figure 3E). Since the surface potential is expected to be altered by the PipX mutation R35A, and subsequently restored by the PII mutation D14A, the results support the importance of electroneutrality for PlmA binding to the PII-PipX complex around residue R35 of PipX. A similar explanation may be provided for the PII^I18A^/PipX^R35A^ pair (Figure 3D), restoring interaction signals to a smaller extent. In this case the side chain of I18 shields a buried lysine that would expose its positive charge in the PII^I18A^ protein (Figure 3E). The increased positive surface potential could be reverted by the R35A change. Less obvious are the compensatory effects provided by the PII^E44A^/PipX^R35A^ and PII^S49A^/PipX^R35A^ pairs, both affecting important residues from the PII T-loop. Interestingly, the three mutations at the C-terminal helices of PipX can be compensated to certain extents by mutation I18A at PII, as showed by the interaction signals from PII^I18A^/PipX^L65Q^, PII^I18A^/PipX^2−70^, or (to a lesser extent) PII^I18A^/PipX^L80Q^ pairs. In the light of the latter results, it is tempting to propose that mutations preventing the “flexed” conformation of PipX improve PlmA binding in the presence of PII^I18A^.

### PlmA is a hallmark of cyanobacteria

Members of the widely distributed GntR-family of transcriptional regulators are classified into several subfamilies according to their diverse C-terminal dimerization/ligand-binding domain (AraR, DevA, FadR, HutC, MocR/GabR, PlmA, and YtrA families). A phylogenetic tree based on 234 PlmA orthologs and representative sequences from the other 6 GntR-subfamilies is shown in Supplementary Figure S1.

**Figure 4:**
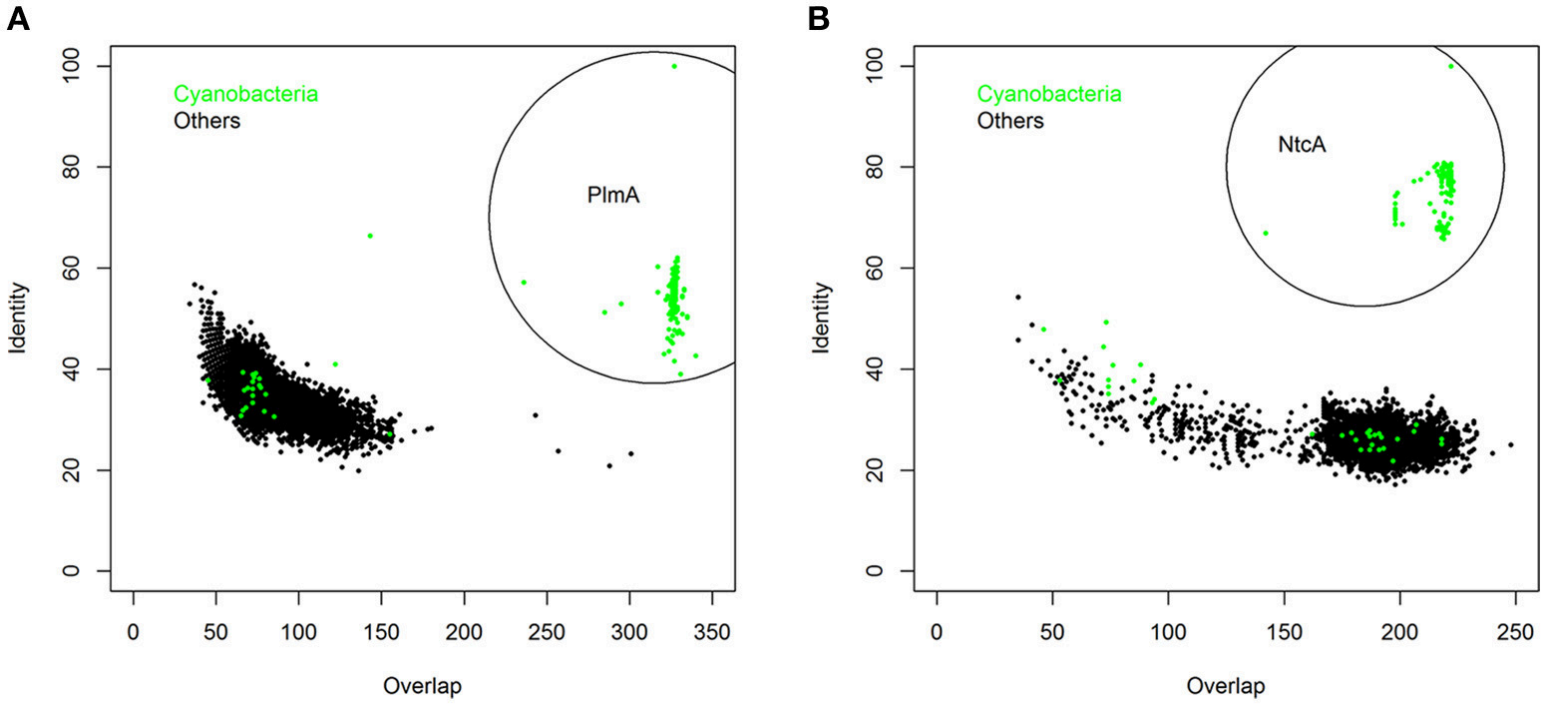
**The PlmA (A) and NtcA (B) subfamilies**. 102,738 and 33,903 sequences homologous to PlmA and NtcA, respectively, were retrieved from the Refseq genomic bacterial database (http://www.ncbi.nlm.nih.gov/refseq) after bidirectional Blast with *S. elongatus* PlmA and NtcA sequences as queries. Horizontal and vertical axes represent, respectively, the amino acid overlap and identity of each hit. Green dots, accounting for 264 (in **A**) and 285 (in **B**) hits, were retrieved from cyanobacterial genomes. Those corresponding to cyanobacterial PlmA and NtcA orthologs (234 and 238 hits, respectively) are encircled.

A bidirectional blast of the Genebank with the *S. elongatus* PlmA sequence as query retrieved 102,743 hits from 23,029 bacterial species or strains, of which 234 were cyanobacteria (Figure 4A). Only cyanobacterial sequences showed both an overlap of ≥200 amino acids and an identity ≥38% with the query sequence. Importantly, the 234 *bona fide* PlmA hits retrieved all the cyanobacterial species included in the analysis, indicating that PlmA is always present in cyanobacteria. Twenty two cyanobacteria (among them *S. elongatus*) had one or more additional GntR family regulators different from PlmA (for example, a MocR/GabR regulator in *S. elongatus*). The analysis confirmed that PlmA forms a distinct subfamily of GntR regulators sharing very limited sequence homology with the other subfamilies.

**Figure 5:**
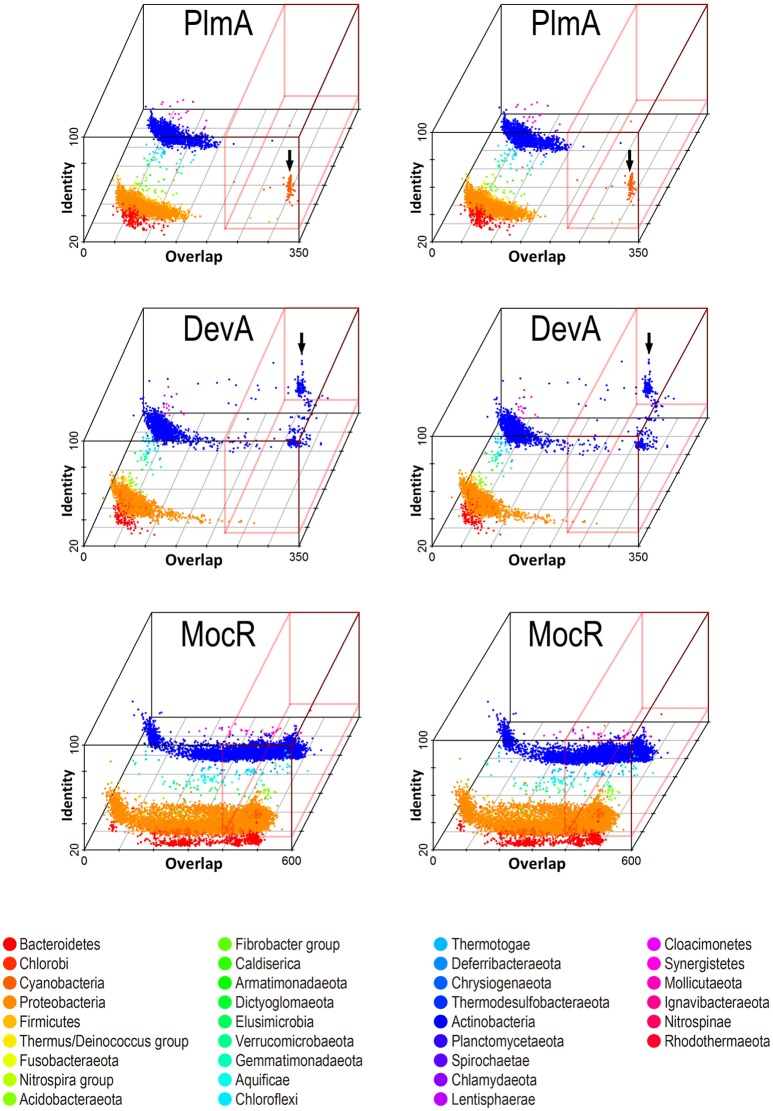
**Stereo view allowing 3D visualization of the phylogenetic distribution of members of GntR subfamilies PlmA, DevA, and MocR**. Representative sequences of each subfamily were used as queries in bidirectional blast searches against the Refseq bacterial database (see Methods). The overlap and identity of the hits (relative to the bait sequence) are shown with a color-code and separated by a third axis according to taxonomy (phylum level). Hits with high overlap and identity are in a red box and arrows point to cyanobacteria and actinobacteria for PlmA and DevA, respectively.

In addition to its universal presence within cyanobacteria, PlmA is also restricted to this phylogenetic group (Figures 4A, 5, top panel, marked with an arrow). This parallels the case of NtcA (Figure 4B), a protein belonging to the CRP family of transcriptional regulators (Herrero et al., 2001). The other GntR regulator with a relatively narrow distribution restricted to a particular *phylum* is DevA from actinobacteria (Figure 5, middle panel, arrow-marked), where it plays specific developmental roles (Hoskisson et al., 2006). The other members of the GntR-superfamily show wider phylogenetic distributions, as shown for MocR in Figure 5. Thus, like the paradigm represented by NtcA, the unique and exclusive distribution of PlmA within cyanobacteria supports the notion that PlmA has unique functions, probably to cope with metabolites relevant to bacteria that perform oxygenic photosynthesis.

### PlmA is essential in *S. elongatus*

**Figure 6:**
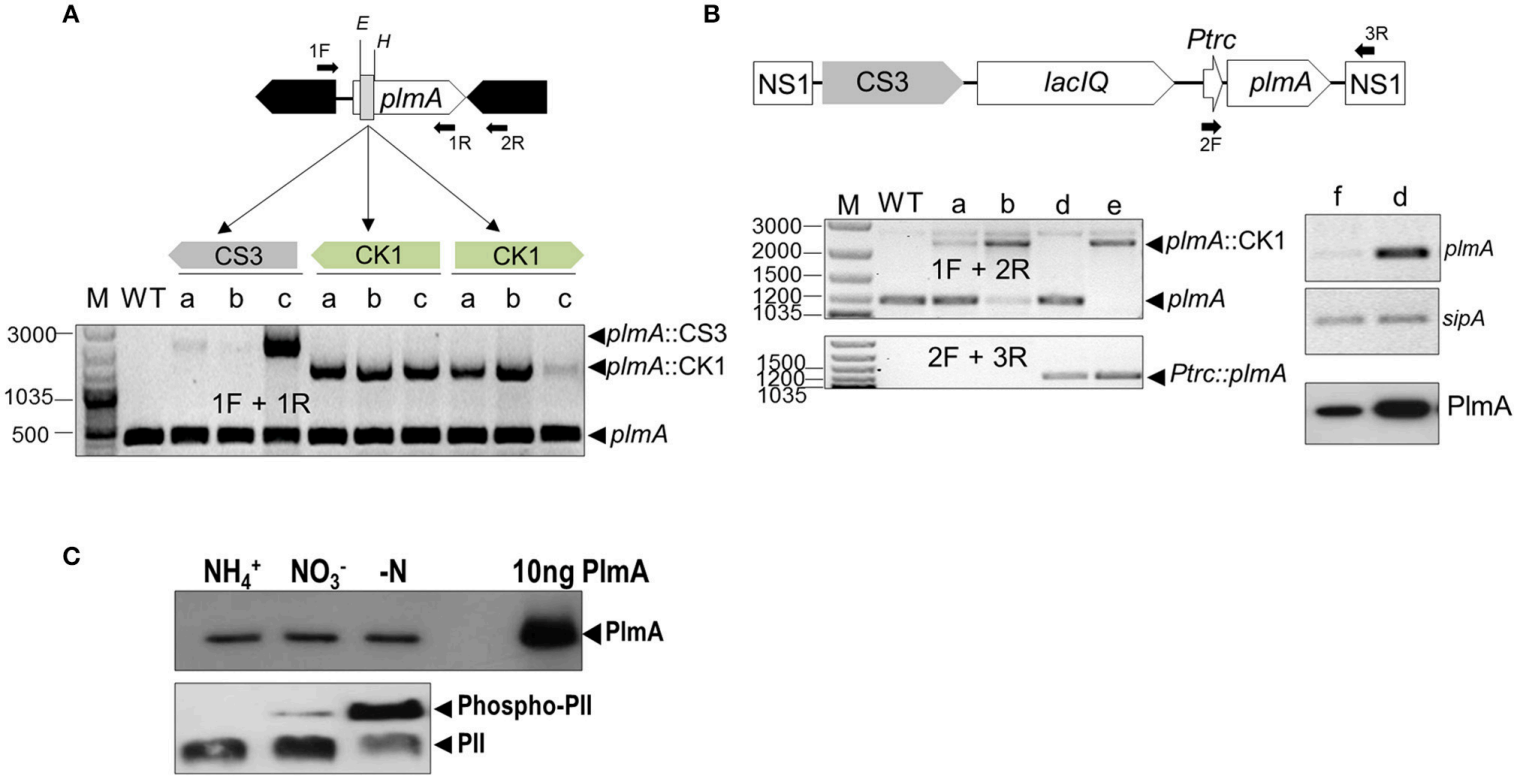
**The *plmA* gene is essential in *S. elongatus***. **(A)** Schematic representation of the *plmA* genomic region with indication of the site of insertion of C.K1 or C.S3 cassettes into the *Eco*RV-*Hinc*II sites, resulting in an internal deletion indicated by a gray rectangle. For each of the indicated cassettes and orientation, three transformant clones (a, b, and c) were PCR-analyzed alongside parental *S. elongatus* (WT) to detect wild type and mutant alleles. **(B)** Ectopic expression of *plmA* allows inactivation of the wild type locus. Top, schematic representation of the region engineered to provide expression of *plmA* from a *Ptrc* promoter. Down-left panels, PCR analysis to detect the indicated *plmA* alleles before (WT) or after transformation with *plmA*::CK1 of WT (lanes a and b), 1Ptrc-PlmA (lane d) and 1Ptrc-PlmA PCK1 (lane e). Down-right panels, RT-PCR assays showing expression of *plmA* and a constitutive gene as control (*sipA*) from 1Ptrc-PlmA (lane d) and 1Ptrc (lane f) strains followed by western-blot to immunodetect the PlmA protein. Positions of relevant PCR primers are indicated as solid arrows, reference size bands (bp) are indicated at the left, and the names of relevant alleles, transcripts or protein to the right of panels. Primers: 1F (plmA-inact-1F), 1R (plmA-YTH-1R), 2R (plmA-inact-1R), 2F (PTRC99Aseq-F), and 3R (NS1-1F). **(C)** Detection of PlmA (top) and PII (bottom) on extracts of *S. elongatus* cells grown under different nitrogen regimens after, respectively, SDS-PAGE and Phos-Tag-SDS-PAGE. The same amount of protein extract was loaded in each well and immunodetection of pure PlmA was carried out as positive control. Immunodetected bands are labeled.

To get insights into PlmA roles in *S. elongatus*, we attempted to inactivate the *plmA* gene by allelic replacement using cassettes providing streptomycin (CS3) or kanamycin (CK1) resistance. The strategy used is schematically represented in Figure 6A. To take into account possible polar effects, the CK1 cassette was inserted in two different orientations [alleles *plmA*::CK1(+) and *plmA*::CK1(-)]. In all cases antibiotic-resistant clones were obtained when *S. elongatus* was transformed with each type of inactivation constructs. However, after several consecutive transfers onto selective plates, all clones remained heteroallelic for the *plmA* gene (Figure 6A), suggesting that *plmA* is essential in *S. elongatus*.

To confirm that PlmA function is required for survival of *S. elongatus* under standard laboratory conditions, we next complemented mutant alleles by ectopically expressing the *plmA* gene (Figure 6B). A copy of *plmA*, fused to the IPTG inducible promoter Ptrc, was introduced into a neutral site (NS1), giving strain 1Ptrc-PlmA. Subsequent RT-PCR and western blot analyses confirmed high levels of expression of *plmA* transcripts and protein (PlmA) in the absence of IPTG, a result in line with previous reports indicating that the Ptrc promoter is very leaky in *S. elongatus* (Espinosa et al., 2015). Next, the null *plmA::*CK1(-) allele was introduced into 1Ptrc-PlmA and complete segregation of kanamycin-resistant transformants (strain 1Ptrc-PlmA PCK1) was verified by PCR. Therefore, the complementation test confirms that *plmA* is part of the essential gene set of *S. elongatus* (Rubin et al., 2015). However, this is not the case with *Synechocystis* sp. PCC 6803 (Fujimori et al., 2005) or *Anabaena* sp. PCC 7120 (Lee et al., 2003), for which successful inactivation of *plmA* genes have been reported.

The differences found within cyanobacteria for the dispensability of the *plmA* gene is reminiscent of the scenario for *glnB* or *ntcA* genes (García-Domínguez et al., 2000; Espinosa et al., 2009). In some cases, inactivation of essential genes may be facilitated by suppressor mutations, as it is the case with *glnB* in *S. elongatus*, which can be readily inactivated in the presence of loss-of-function mutations at *pipX* (Espinosa et al., 2009, 2010). However, attempts to obtain *S. elongatus plmA* null strains in different mutant backgrounds have so far been unsuccessful (data not shown).

Next, we tested whether the *in vivo* levels of the PlmA protein are subjected to nitrogen regulation. To this end, we compared the protein levels in *S. elongatus* cultures grown with ammonium, nitrate or after nitrogen depletion, three conditions that correlate with different levels of PII phosphorylation according to changes in carbon to nitrogen ratios (Forchhammer and Tandeau de Marsac, 1995). As shown in Figure 6C, no differences in PlmA protein levels could be detected amongst the different conditions. Since the relative proportions of PII/Phospho-PII did change as expected, the results clearly indicate that PlmA protein levels are independent of the intracellular 2-OG levels.

### Levels of molecular players of the PII-PipX-PlmA interaction system

**Figure 7:**
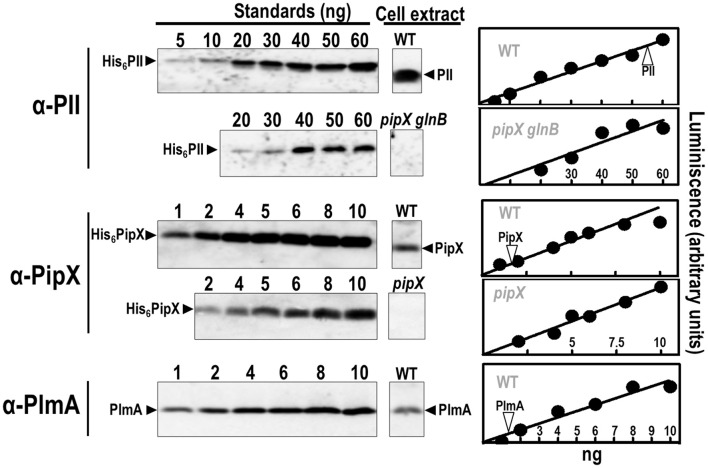
**Immunoquantification of PlmA and related proteins in *S. elongatus***. Representative examples of immunodetection signals with pure recombinant proteins (His_6_-PII, His_6_-PipX, and PlmA) (left panels) and whole cell extracts of wild-type or indicated mutant strains (middle panels) are shown. Quantified signals with increasing amounts of the indicated recombinant proteins were plotted (Right panels) and the signal from extracts was interpolated in the calibration line (position indicated with a vertical arrowtip). Specificity controls of the PipX and PII immunoassays are provided by the null mutants *pipX* and *pipX glnB*, respectively.

To determine the relative proportions in the levels of PII, PipX, and PlmA proteins in *S. elongatus* cells, we performed western blots from ammonium-containing cultures, conditions that would favor PII-PipX complexes. As shown in Figure 7 and summarized in Table 3, PII is ~14-fold more abundant (as moles of polypeptide chains) than PipX, and PipX is ~7-fold more abundant than PlmA. Recently, a whole-cell proteomics study carried out with nitrate-containing cultures of *S. elongatus* (Guerreiro et al., 2014) yielded similar molar ratios to those found by us for PlmA, PII, and PipX (Table 3). The study of Guerreiro et al. calculated the number of PlmA molecules per cell to about 200 dimers (Table 3), a value not far from that for NtcA (~500 dimers), suggesting that the PlmA regulon could be quite large, as is the case for NtcA.

**Table 3:** **Levels of molecular players of the PII-PipX-PlmA system in *S. elongatus***.

	**Level per mg total protein (Western blotting)^a^**		**Relative abundance (as subunits)^b^**	
	**As mass**	**As subunits^c^**	**Western blot^a^**	**Proteomics^d^**
	μg/mg	pmol/mg	%	
PII	2.2 ± 0.6	177	100	100
PipX	0.13 ± 0.04	12	7	7.6
PlmA	0.06 ± 0.02	1.7	1	0.7
NtcA	–	–	–	1.7
NAGK	–	–	–	3.6

a *Western blotting results (mean ± SD) were obtained as illustrated in Figure 7 from at least 2 independent experiments*.

b *The abundance of subunits is given as a percentage of the abundance of PII subunits, which thus is given an arbitrary value of 100*.

c *The level of subunits is that for the mean values of the preceding column. It has been rounded to the closest integer in the cases of PII and PipX and to the closest first decimal in the case of PlmA*.

d *Proteomics data are derived from Guerreiro et al. (2014)*.

Since the ratios between the levels of PlmA, PII, and PipX appear similar between nitrate- and ammonium-containing cultures of *S. elongatus* (Figure 6C and our own data not shown), the work of Guerreiro et al. (2014) is also informative for the proteins that interact with PII or PipX within the nitrogen interaction network. NtcA and NAGK are also present in small molar amounts relative to their respective partners, PipX and PII (NtcA/PipX and NAGK/PII molar ratios, as subunits, 0.22 and < 0.05, respectively). From these data, the known stoichiometry of PII-PipX and NtcA-PipX complexes, and the stoichiometry that we proposed for the PII-PipX-PlmA complex (see below), the probable engagement of these proteins in the different complexes can be inferred.

Under conditions of low 2-OG (high N/C), PII binds to NAGK forming the PII-NAGK complex and to PipX forming the PII-PipX complex, whereas PlmA would bind only to the later complex. The large molar excess of PII over PipX and NAGK and of PipX over PlmA would suggest that PipX, NAGK, and PlmA could exist largely as parts of these complexes. In contrast, much PII will remain free, being available for interaction with additional proteins, including some transporters (Hisbergues et al., 1999; Lee et al., 2000).

Under conditions of nitrogen limitation all PII complexes dissociate, allowing formation of NtcA-PipX complexes. The large pool of (phosphorylated) PII is then available for other protein partners, although no proteins interacting with PII under conditions of nitrogen limitation have been reported in cyanobacteria to date. PipX, more abundant than NtcA, would also be available for interaction with yet unknown partners under these conditions. In turn, PlmA would be released from its ternary complex with PII and PipX, and would also be available for interacting with its putative binding targets.

### An interaction-based model for PlmA function

The identification of PlmA, a second transcriptional regulator involved in the nitrogen interaction network of cyanobacteria is the successful result of the search for proteins interacting with PII-PipX complexes. This search was motivated by the structure of PipX when in complex with PII (Llácer et al., 2010) as well as by the inferred implication of PipX in NtcA-independent transcriptional regulation (Espinosa et al., 2014).

**Figure 8:**
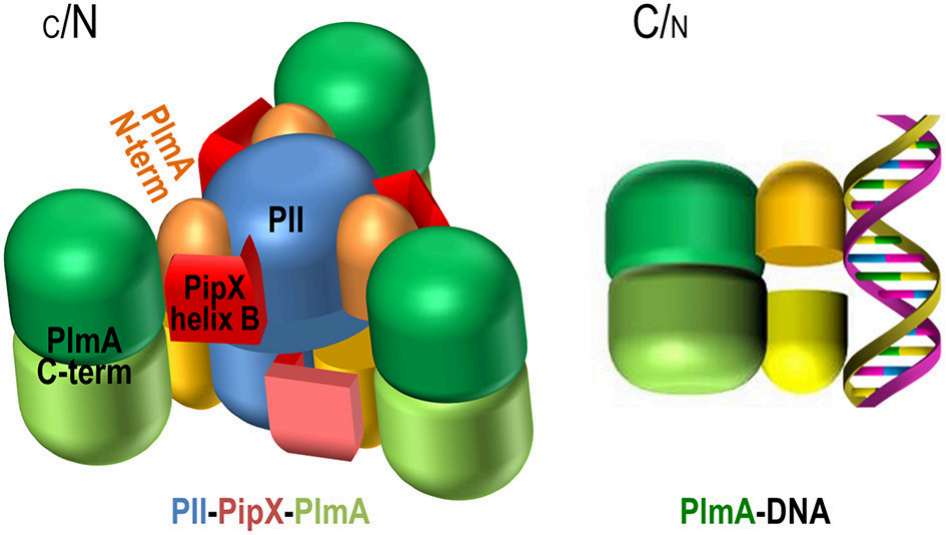
**Representation of the possible structure of the PII-PipX-PlmA complex and model for PlmA regulation according to C/N balance**. PII trimers are blue, C-terminal helices of the PipX molecules (schematized in the extended conformation) are colored in two hues of red, and the N-terminal domains of PlmA dimers are colored yellow or orange, whereas the C-terminal domains are shown in two hues of green. The three-fold axis of the PII-PipX-PlmA complex is vertical and makes an angle of about 10° with the plane of the paper, exiting toward the viewer above the point of crossing with the paper. See text for more details.

The results from extensive and detailed two-hybrid and three-hybrid interaction analyses are consistent with the following interpretations: (a) the C-terminal helices of PipX are directly involved in interactions with PlmA and (b) PII would not provide direct contacts with PlmA. Instead, PII as part of the PipX-PII complexes would facilitate the “extended” conformation of helices A and B of PipX, providing access to the main interaction determinants for PlmA binding. In the light of these findings, of the dimeric nature of PlmA revealed here, and given the need to cope with the symmetry requirements involved in making a monodisperse complex (rather than an open-ended, infinitely growing network) of molecules having three-fold symmetry (PII) and two-fold symmetry (GntR), we propose a model for this complex which is schematically illustrated in Figure 8.

In the proposed PII-PipX-PlmA complex (stoichiometry 6:6:6), two PII-PipX complexes (stoichiometry 3:3) having essentially the same structure that was reported for the PII-PipX complex (Llácer et al., 2010) would be connected by three PlmA dimers. They would have their threefold axes aligned and the convex parts of the trimeric bodies of the two PII molecules looking outwards (up and down if the molecular three-fold axis is placed vertically). The C-terminal helices of PipX would be extended toward the laterally-located PlmA dimers, that would connect the two PII-PipX trimers by interacting with the PipX molecules. The N-terminal DNA-binding domain of PlmA, given its small size, would be embraced by PipX helices, so that the domain from one subunit would be anchored in a PipX molecule from one PII-PipX trimer, and the other domain of the same PlmA dimer would be anchored on a PipX molecule from the other trimer. If follows that sequestration of the DNA binding domains of PlmA would render this transcriptional regulator inactive.

Increasing the levels of 2-OG would release all PII-dependent complexes, preventing activation of NAGK, stimulating NtcA activity (NtcA-PipX complexes), and presumably releasing PlmA, that would then be free to find its DNA targets. Therefore, the model proposes a common regulation by PII/PipX in response to 2-OG (and ATP/ADP) signals for both NtcA and PlmA but with important mechanistic differences: PipX is in one case part of the active transcription factor (NtcA-PipX complexes) and in the other case would be part of the inactive complex with the transcriptional regulator (PII-PipX-PlmA complexes).

Given the important regulatory complexity which is emerging for GntR family members (Edayathumangalam et al., 2013; Blancato et al., 2016; Fillenberg et al., 2016), it is reasonable to assume that additional signals can be integrated into the system via the C-terminal and effector binding domain of PlmA. As it is the case with other GntR family members, PlmA could detect specific and yet unknown chemical signals via this domain, which would be accessible when in complex with either PII-PipX or DNA (Figure 8) and thus the possibility of PlmA responding to environmental clues other than 2-OG and ATP/ADP should be considered in future studies.

## Author contributions

JL, JE, AC, and VR designed research; JL, AO, PS, AF-N, and LT performed research. AC and VR wrote the paper. All authors analyzed data.

## Funding

This work was supported by grants BFU2015-66360-P to AC and BFU2014-58229-P to VR from the Spanish Ministry of Economy and Competitivity. AO was the recipient of Grisolia Fellowship from Consellería d'Educació of the Valencian Government and AF-N and LT held FPI fellowships/contracts from Ministry of Economy and Competitivity. JE and VR were supported by grants GV/2014/073 and PrometeoII/2014/029, respectively, from the Consellería d'Educació of the Valencian Government.

### Conflict of interest statement

The authors declare that the research was conducted in the absence of any commercial or financial relationships that could be construed as a potential conflict of interest. The reviewer KF declared a past co-authorship with one of the authors VR to the handling Editor, who ensured that the process met the standards of a fair and objective review.
