# Supplementary figures and images for: Expanding the Cyanobacterial Nitrogen Regulatory Network: The GntR-Like Regulator PlmA Interacts with the PII-PipX Complex
Source: Front Microbiol. 2016 Oct 28;7:1677. doi: 10.3389/fmicb.2016.01677 (PMC5083789; doi:10.3389/fmicb.2016.01677)

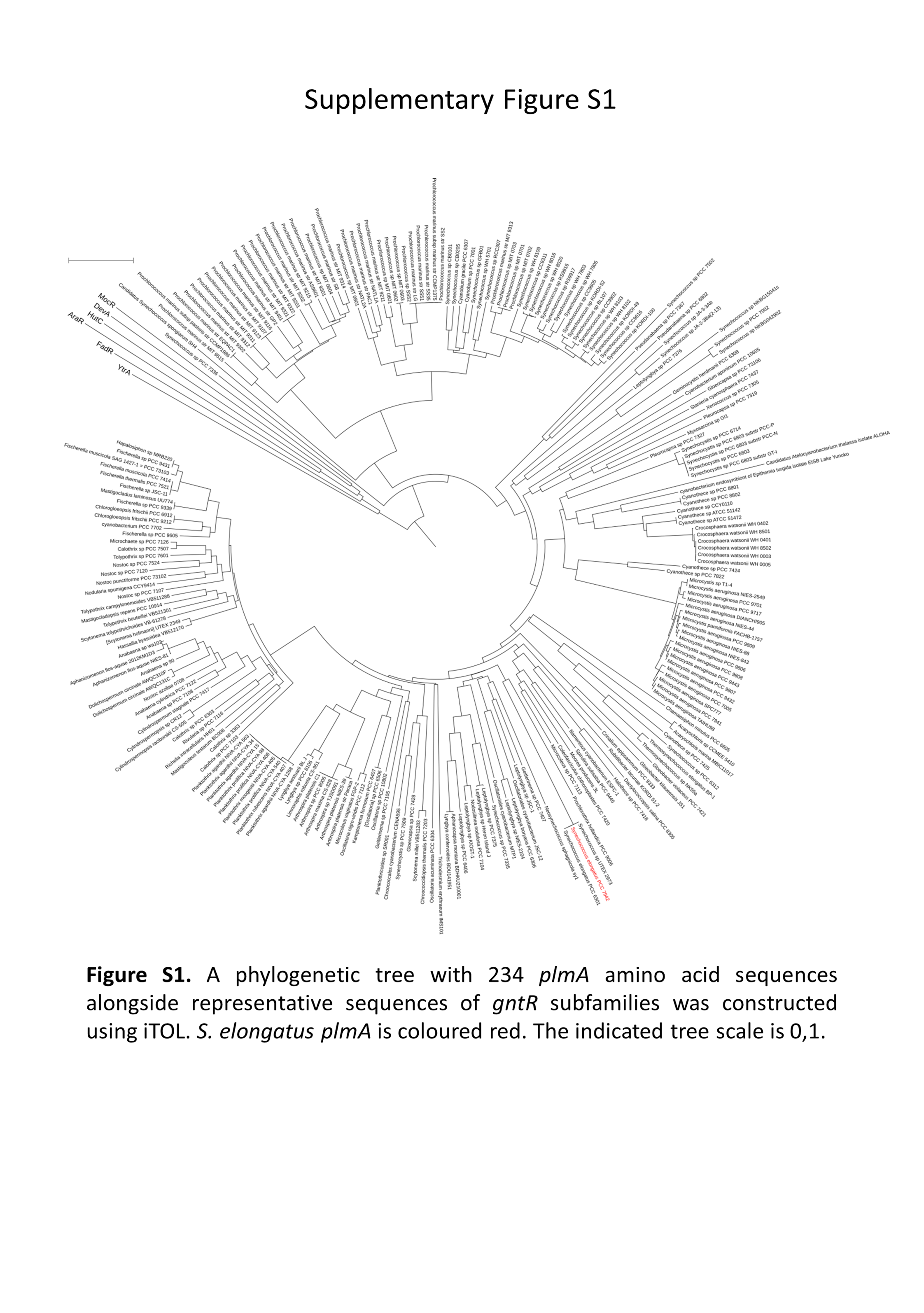

Supplement: Supplementary file 1 [file Image1.TIF]
